# The inter- and multi- generational epigenetic alterations induced by maternal cadmium exposure

**DOI:** 10.3389/fcell.2023.1148906

**Published:** 2023-04-20

**Authors:** Lauren Lawless, Linglin Xie, Ke Zhang

**Affiliations:** ^1^ Institute of Bioscience and Technology, Texas A&M University, Houston, TX, United States; ^2^ Department of Nutrition, Texas A&M University, College Station, TX, United States

**Keywords:** cadmium, epigenetics, placenta, fetal development, maternal nutrition

## Abstract

Exposure to cadmium during pregnancy, from environmental or lifestyle factors, has been shown to have detrimental fetal and placental developmental effects, along with negatively impacting maternal health during gestation. Additionally, prenatal cadmium exposure places the offspring at risk for developing diseases in infancy, adolescence, and adulthood. Although given much attention, the underlying mechanisms of cadmium-induced teratogenicity and disease development remain largely unknown. Epigenetic changes in DNA, RNA and protein modifications have been observed during cadmium exposure, which implies a scientific premise as a conceivable mode of cadmium toxicity for developmental origins of health and disease (DOHaD). This review aims to examine the literature and provide a comprehensive overview of epigenetic alterations induced by prenatal cadmium exposure, within the developing fetus and placenta, and the continued effects observed in childhood and across generations.

## 1 Introduction

Cadmium is a naturally occurring metallic element used in many industrial processes, such as electroplating, galvanizing, producing batteries and solar panels, and zinc and iron smelting (Johri et al., 2010; Ramos-Ruiz et al., 2017). It is distributed widely in the environment, due to the rise of global industrialization, and is highly concentrated in cigarette smoke (Järup et al., 1998; Satarug et al., 2017). Individuals can become exposed to cadmium through the consumption of crops grown in contaminated soil and polluted seafood, as well as occupation or habitation in dense industrial areas and residences near toxic waste dump sites (Aoshima, 1987; Pizzol et al., 2014). Overconsumption or inhalation of this metal has been shown to induce genotoxicity, ROS production, and apoptosis, further leading to the development of cardiovascular disease, renal dysfunction, and carcinogenesis (Satoh et al., 2002; Waalkes, 2003; He et al., 2006). Moreover, cadmium exposure can disrupt essential metal ion concentrations, further leading to metabolic disruption, insulin resistance, and obesity (Jackson et al., 2022).

Cadmium has also been identified as an endocrine disruptor, making it particularly dangerous during prenatal exposure. Endocrine-disrupting chemicals can stimulate or inhibit hormone production, alter hormone transport throughout the body, and interfere with normal reproductive function (Vaiserman, 2014). Prenatal cadmium exposure has been shown to interfere with progesterone, testosterone, and leptin synthesis, which alters offspring’s thyroid function and the development of their reproductive systems (Iijima et al., 2007; Ishitobi et al., 2007; Stasenko et al., 2010; Samuel et al., 2011; Banzato et al., 2012). Furthermore, prenatal cadmium exposure has been linked to spontaneous abortions and premature delivery (Yang et al., 2006). Fetal growth restriction is also a common manifestation among offspring exposed to gestational cadmium, evidenced by decreased birth weight and height, and a reduced head circumference (Zhang et al., 2018). Additionally, cadmium exposure during pregnancy leads to a disturbed translocation of placental metal ions, such as zinc, and reduces the maintenance of fetal nutrition and viability, which negatively impacts fetal growth and development (Mikolić et al., 2015). Moreover, prenatal cadmium exposure has been associated with the development of offspring diseases as they reach adulthood. A mouse model revealed that prenatal cadmium exposure induced hyperglycemia as the offspring mice reached puberty and impaired glucose tolerance in adulthood (Yi et al., 2021). Increased maternal blood and hair levels of cadmium were also associated with an increased incidence of congenital heart defects, which significantly predisposes the offspring to poor cardiac outcomes and the development of cardiovascular disease in adulthood (Jin et al., 2016; Ou et al., 2017; Bokma et al., 2018; Bauer et al., 2019).

Recently, investigating the developmental origins of health and disease (DOHaD) has been given much attention. This theory, known as the Barker hypothesis, states that adverse prenatal and early life factors, such as poor nutrition or lifestyle influences, significantly impact fetal and childhood growth, and predispose the offspring to metabolic syndrome, subsequently leading to the onset of adolescent and adult diseases (Barker et al., 2002; Edwards, 2017). Mechanistically, epigenetic alterations highlight these adverse disease outcomes. Due to environmental or maternal lifestyle factors, these modifications have been shown to significantly induce offspring metabolic syndrome, obesity, heart disease, and hypertension (Ryznar et al., 2021). An accumulation of data has demonstrated that prenatal cadmium exposure induces many epigenetic alterations, such as DNA methylation and post-translational histone modifications, as well as influences on differential micro-RNA expression within the developing offspring (Vilahur et al., 2015). This review aims to summarize recent research findings regarding the effects of gestational cadmium exposure on epigenetic variations within both the placenta and fetus, as well as to examine how these epigenetic alterations influence the clinical outcomes of the next generations in both childhood and adult stages.

## 2 Cadmium-associated epigenetics alterations during pregnancy

Epigenetics is defined as the dynamic changes to DNA to affect gene expression apart from alterations to the underlying sequence (Berger, 2007). Examples of epigenetic alterations include DNA methylation, posttranslational modifications of histone proteins, and small non-coding RNA molecules that can interfere with gene expression (Vilahur et al., 2015). Modification of epigenetic patterns has been found to result from environmental and lifestyle stimulants, such as exposure to ecological contaminants, poor nutrition, obesity, and smoking. Furthermore, prenatal exposure to such environmental factors can impose an adverse uterine environment, predisposing the developing offspring to aberrant epigenetic alterations and increasing disease risk.

### 2.1 Cord blood

As previously mentioned, cadmium exposure has been shown to induce many epigenetic modifications during prenatal development. Maternal blood cadmium concentrations were found to be associated with genomic DNA hypomethylation of the gene, ATP9A, and variable methylations of the gene cg24904393, within the umbilical cord blood of human infants (Park et al., 2022). ATP9A is important for phospholipid transporting ATPase activity. Decreased expression of this gene has been shown to increase extracellular vesicle release and apoptosis, as well as interrupt the recycling process of critical transport proteins, such as GLUT-1 (Park et al., 2022). Additionally, the gene, cg24904393, encodes the plasminogen protein, which is vital in blood coagulation and fibrinolysis (Park et al., 2022). Dysregulation of this gene can increase the risk of thrombosis, which proves to be detrimental to the developing offspring (Park et al., 2022). Moreover, within the cord blood of cadmium-exposed infants, hundreds of cadmium-associated differentially methylated regions (DMRs) were identified. These DMRs were most commonly located within the maternal imprinting control regions. The top three functional categories associated with the cadmium-induced methylation modifications include BMI regulation, atrial fibrillation, and hypertension (Cowley et al., 2018).

### 2.2 Placental development

The placenta is an organ that exists during pregnancy to modulate nutrient, oxygen, and waste exchange from the mother to the fetus. Maternal spiral artery remodeling is an essential event in placental development that allows for sufficient blood to be delivered from the maternal circulation to the placenta (Woods et al., 2018). Concurrently, extensive villous branching and vascularization ensure to establish a highly specialized network of maternal blood and fetal capillaries within the placental layer adjacent to the fetus (Rossant and Cross, 2001). Proper development of this organ is vital to support the growth of the fetus, as obstructions in its formation result in impaired nutrient transport, leading to fetal growth restriction and maternal complications of pregnancy, such as pre-eclampsia (Wier et al., 1990; Wang et al., 2016a; McKinney et al., 2016; Zhang et al., 2016; Hung and Chen, 2018; Rana et al., 2019; Tenório et al., 2019).

Prenatal cadmium exposure has been shown to induce many different epigenetic alterations within the placenta, which significantly impact its function. In a human study of mother-infant pairs, placental cadmium levels were significantly associated with sex-specific DNA methylation changes within this organ. Within the female offspring, differential methylation was observed near transcriptional start sites for cell damage response genes, whereas methylation changes were observed in genes involved in placental development, cell differentiation, and angiogenesis in the males (Mohanty et al., 2015). Additionally, increased maternal cadmium levels were significantly associated with decreased DNA methylation within the promoter region of *PCDHAC1* in the human placenta, which is a gene belonging to the protocadherin gene family and is very important for fetal growth (Everson et al., 2016). There was also a strong association found between decreased DNA methylation of placental *PCDHAC1* and an increased odds of small for gestational age (SGA) offspring and decreased head circumference upon prenatal cadmium exposure in this study (Everson et al., 2016). In mice, prenatal cadmium exposure was found to decrease the expression of placental GLUT-3, due to site-specific DNA methylation, consequently leading to fetal growth restriction (Xu et al., 2016). Moreover, the placenta is an organ rich in the expression of imprinted genes, which are genes defined by their preferential expression from one of the two parental alleles and are regulated by epigenetic marks responding to environmental stimuli (Xu et al., 2017; Cowley et al., 2018). Consistently, the maternal imprinted gene, *Cdkn1c*, was significantly upregulated, while the paternally imprinted gene, *Peg10*, was significantly downregulated in the cadmium-exposed mouse placenta, and these changes were associated with restricted offspring growth. This was found to be a consequence of a decreased methylation level in the promoter region of *Cdkn1c* and an increased methylation level within the promoter region of *Peg10* (Xu et al., 2017). These genes are important for nutrient transport and placentation, as differential expression of *Cdkn1c* and *Peg10* were associated with discrepancies in cell proliferation and survival, and fetal viability and labyrinth malformation, respectively (Koppes et al., 2015; Xu et al., 2017). Recently, the expression of *Cdkn1c* was found to be upregulated in the cadmium-exposed mouse placenta of both male and female offspring and was associated with decreased fetal growth (Simmers et al., 2022). The female offspring displayed no significant differences between the ratio of *Cdkn1c* transcripts derived from the maternally and paternally inherited alleles, while the male offspring had a significant increase in the expression percentage of *Cdkn1c* from maternally inherited alleles, indicating no loss of imprinting (Simmers et al., 2022). On the contrary, this increased expression of *Cdkn1c* in the placentas was not found to be a result of the differential DNA methylation, as discussed previously, since there were no changes in the methylation profile within the promoter region upon cadmium treatment (Simmers et al., 2022). Instead, the increased expression of *Cdkn1c* in this study was found to be a consequence of altered placenta morphology (Simmers et al., 2022). The differences in these epigenetic alterations could potentially be attributed to inconsistent study designs, as cadmium provided 5 weeks prior to pregnancy, during mating, and throughout pregnancy did not induce methylation differences, while decreased DNA methylation of *Cdkn1c* was observed when cadmium was provided at E7.5 (Xu et al., 2017; Simmers et al., 2022).

MicroRNAs (miRNAs) are epigenetic modifiers that are important for gene expression. Within the placenta, miRNAs are involved in regulating trophoblast differentiation, migration, invasion, and vasculogenesis (Mouillet et al., 2015; Hayder et al., 2018). However, exposure to environmental factors, such as cadmium, can induce alterations in the expression of these placental miRNAs, leading to changes in the regulation of genes involved in proper placental development and, subsequently, maternal complications of pregnancy, like preeclampsia, fetal growth restriction, and preterm birth (Kotlabova et al., 2011). Prenatal cadmium exposure has been found to significantly increase the expression of miR-509-3p and miR-193b-5p within the human placenta, which may affect both placental function and nervous system development (Tehrani et al., 2022). miR-509-3p is negatively associated with cell migration and invasion, two events that are vital for proper spiral artery remodeling in placental development (Su et al., 2015; Pan et al., 2016; Ahir et al., 2017). Consistently, increased miR-193b-5p expression is associated with cases of preeclampsia and fetal growth restriction due to hindered trophoblast migration and invasion (Zhou et al., 2016; Awamleh et al., 2019; Östling et al., 2019; Awamleh and Han, 2020). Furthermore, cadmium treatment of JEG-3 cells showed a significant decrease in cell migration, due to increased expression of the TGF-β pathway family members. These findings were also associated with altered miR-26a expression, indicating that cadmium modulates placental miRNAs, contributing to increased activation of TGF-β signaling and abnormal trophoblast migration (Brooks and Fry, 2017).

### 2.3 Fetal growth

Prenatal cadmium exposure has also been shown to induce many epigenetic alterations within the developing fetus. During early development, an increased occurrence of embryonic death, fragmentation, and developmental blockades upon cadmium treatment was observed in mice (Zhu et al., 2021). Interestingly, the surviving embryos experienced epigenetic changes at the 8-cell stage, including histone acetylation, evidenced by increased histone deacetylase 1, and genomic DNA methylation, manifested by *H19* hypomethylation (Zhu et al., 2021). *H19* is a gene involved in early embryonic development and implantation, along with increased ROS levels and DNA damage (Zhu et al., 2021). Additionally, differential DNA methylation was observed in dozens of genes relating to fetal gene expression, tissue morphology, cancer, lipid metabolism, and apoptosis within the cadmium-exposed infant (Sanders et al., 2014).

Although prenatal cadmium exposure has been shown to induce many different epigenetic alterations, there are inconsistencies among the modifications reported, leading some to suggest sex as a factor in cadmium-induced toxicities. In a human study, Kippler et al. (2013) reported that male offspring exhibited global hypermethylation, specifically of genes related to cell death pathways, within cord blood DNA, while the females showed global hypomethylation of genes associated with organ development, morphology, and bone mineralization (Kippler et al., 2013). These results provide compounding evidence to previous reports, in which fetal head and femur length in girls is decreased upon prenatal cadmium exposure and cadmium-associated osteoporosis and fractures are particularly observed in women (Engström et al., 2012; Kippler et al., 2012). Nevertheless, several of the individual CpG sites that were positively associated with cadmium were inversely correlated with birth weight, indicating that cadmium-induced fetal growth restriction potentially occurs through differential DNA methylation (Kippler et al., 2013). Additionally, cadmium-exposed female infants exhibited decreased birth weights, accompanied by hypomethylation of *PEG3*, which is a paternally expressed imprinted gene that encodes a zing-finger protein that plays a role in p53-mediated apoptosis (Deng and Wu, 2000; Vidal et al., 2015). The cadmium-exposed male newborns were significantly lighter, but *PEG3* methylation was not affected (Vidal et al., 2015). Interestingly, female offspring of cadmium-exposed women with higher zinc levels showed increased methylation of *PEG3* when compared to those offspring of zinc-deficient mothers, indicating that adequate zinc intake may mitigate these cadmium-induced epigenetic alterations (Vidal et al., 2015). Furthermore, prenatal cadmium exposure in rats resulted in elevated expression of DNMT3A in the livers of the male offspring and a decreased expression within the female offspring. DNMT3A is involved in *de novo* CpG methylation (Castillo et al., 2012). This finding corresponded with hypermethylation of the glucocorticoid receptor within the male offspring and hypomethylation within the females (Castillo et al., 2012). This could induce altered glucocorticoid metabolism, which is significantly linked to an increased risk of cardiometabolic disorders in adulthood (Castillo et al., 2012).

## 3 Cadmium-associated epigenetic alterations during childhood

It has been speculated that many cadmium-associated epigenetic changes within the fetal stage of development contribute to adulthood diseases. A number of studies have employed an experimental design that includes follow-up data collection in the child and adulthood stages to investigate the status of the epigenetic alterations. As previously mentioned, prenatal cadmium exposure induces DNA methylation modifications within the developing fetus. Interestingly, Kippler et al. (2013) reported that similar methylation profiles about offspring growth observed in newborn cord blood were continually observed in children at 4.5 years old, along with the growth effects induced by prenatal cadmium exposure (Kippler et al., 2013). Furthermore, a higher maternal urinary cadmium concentration was significantly associated with a slower weight gain from age 3 months to 4 years, indicated by slower height and BMI trajectories (Chatzi et al., 2019). This effect was seemingly stronger in girls, specifically in those offspring exposed to cadmium during the first trimester, a period in which epigenetic remodeling is highly active (Chatzi et al., 2019). A recent study investigated if the epigenetic changes associated with prenatal cadmium exposure persist from birth into childhood. It reported several DNA methylation differences within the cord blood that appeared to still be associated with prenatal cadmium exposure at 9 years of age. Interestingly, these epigenetic changes were found to mainly be a consequence of gestational cadmium exposure rather than long-term childhood cadmium exposure (Gliga et al., 2022). However, this study was unable to significantly detect specific DNA methylation changes that persist from birth to prepubertal age due to small power and too few overlapping differentially methylated positions and regions to perform enrichment and pathway analyses, and it was unable to control for other environmental exposures and lifestyle factors (Gliga et al., 2022). Nevertheless, this data highlights the importance of determining gestational offenses as origins of disease rather than life-long exposure alone.

## 4 Cadmium-associated multigenerational epigenetic effects

During embryogenesis, the developing offspring experience two major cycles of epigenetic reprogramming. The first occurs during the preimplantation stage and the second during germ cell development (Li et al., 2019). During the preimplantation stage, the paternally inherited genome undergoes global DNA demethylation, followed by subsequent cell divisions to further induce the loss of epigenetic modifications (Sinclair et al., 2007). Interestingly, within the mouse, the maternally derived genome was found to retain its methylation patterns at this stage (Santos et al., 2005). As development continues into gastrulation and germ cell specification, both imprinted and non-imprinted genes are dynamically demethylated, ensuring that the inherited epigenetic alterations are deleted within the germline (Sinclair et al., 2007). This erasure of the gametic epigenetic patterns allows for the embryo to establish its own epigenetic profile indispensable for proper development (Huntriss, 2021). However, there are reports of genes in primordial germ cells escaping demethylation, thereby carring the epigenetic markers to F2 generation (Seisenberger et al., 2012; Hackett et al., 2013; Heard and Martienssen, 2014). Recently, transgenerational epigenetic inheritance was demonstrated, as two metabolic-related genes were specifically methylated and silenced in mouse embryonic stem cells, inducing abnormal metabolic phenotypes (Takahashi et al., 2023). Interestingly, these methylation and phenotypic changes were retained and transmitted across multiple generations, providing evidence contrary to the widely accepted notion that epigenetic patterns are erased during embryogeneis (Takahashi et al., 2023). Nevertheless, because the window of epigenetic reprogramming occurs very early in gestation, proceeding placentation and organogenesis, the embryo is vulnerable to epigenetic alterations caused by environmental stimuli, such as cadmium exposure, which can increase the risk of congenital defects and subsequent diseases in later life (Joubert et al., 2012; Manikkam et al., 2012; Vaiserman, 2014; Vilahur et al., 2015). The multigenerational effect of cadmium induced epigenetic alterations, though, remains controversial and poorly understood.

Cadmium has been shown to impact protein acetylation in testicular development. Briefly, lysine acetylation, specifically of histone H4, is an important step in the formation of sperm cells, as it is a prerequisite for histones to be replaced by transition nuclear proteins in spermatogenesis (Awe and Renkawitz-Pohl, 2010). Lysine succinylation is another post-translational modification that plays a regulatory role in cell differentiation and organ development (Xie et al., 2012). Cadmium exposure was found to impact GAPDH activity, and ATP and cAMP levels within germ cells in mice, which then further inhibited lysine acetylation and succinylation within the testes, resulting in reproductive injuries (Yang et al., 2018). Additionally, acetylation of histone H4K5 and H4K12 was also inhibited by cadmium treatment, further inducing spermatogenesis failure (Yang et al., 2018). These findings were also consistent with other reports of protein post-translational modifications impacting sperm function. Cadmium exposure to spermatozoa, *in vitro*, exhibited impacted GAPDH and AMPK activity and ATP production, further leading to inhibited sperm motility. This was found to be a result of increased tyrosine phosphorylation (Wang et al., 2016b). Consistently, cadmium exposure in mice also induced dihydrolipoamide dehydrogenase tyrosine phosphorylation, which significantly impacted the activity of the TCA cycle and, subsequently, oxidative phosphorylation, leading to decreased ATP production and poor sperm motility within the developing testes (Li et al., 2016).

A rat model demonstrated that gestational cadmium exposure induced decreased levels of progesterone in offspring ovarian granulosa cells, due to significant decreases in the mRNA levels of the steroidogenic enzymes, StAR, and Cyp11a1, at post-natal day 56 (Liu et al., 2020). This gene expression decrease was found to be associated with an upregulation of the miRNAs, miR-27a-3p and miR10b-5p, within the ovarian granulosa cells (Liu et al., 2020). miR-27a-3p regulates estrogen and progesterone receptors’ expression and mediates these hormones’ metabolism (Liu et al., 2020). miR-10b-5p acts directly on StAR and mitigation of its expression can induce reproductive damage (Liu et al., 2020). Interestingly, decreased progesterone production and StAR expression were continually observed in the F2 rat offspring, indicating that prenatal cadmium-associated epigenetic alterations could have a transgenerational effect (Liu et al., 2020). Moreover, the apoptotic gene, Bcl2, was significantly altered in the ovarian granulosa cells of F1 and F2 rat offspring, accompanied by increased apoptotic cell bodies (Liu et al., 2021). This was accompanied by differential expression of miR-16-5p and miR92a-2-5p, which are regulators of Bcl2 expression (Liu et al., 2021). This indicated that prenatal cadmium exposure dysregulated the expression profile of these miRNAs within the ovarian granulosa cells, thereby leading to increased cell death through modulation of Bcl2, in both F1 and F2 offspring (Liu et al., 2021). Another recent study investigated the multigenerational effects of prenatal cadmium exposure on testicular function. The F1 and F2 male mice exhibited immature Sertoli and Leydig cells (Huang et al., 2020). Additionally, the F1 mice showed detachment of spermatogonia from the basement membrane, and the tubular diameter was impacted in both the F1 and F2 mice, indicating impacted spermatogenesis (Huang et al., 2020). Prenatal cadmium exposure also affected the secretion of male hormones, as a gonadotropin-releasing hormone, luteinizing hormone, progesterone, and testosterone were all significantly decreased in the F1 mice, while testosterone was significantly increased in the F2 mice (Huang et al., 2020). These results were found to be a result of differential expression of the steroidogenic enzymes, SF-1 and StAR, within both the F1 and F2 mice (Huang et al., 2020). These enzymes were regulated by the cadmium-induced expression of miR-328a-5 and miR-10b-5p, respectively (Huang et al., 2020). When the miRNA expression was increased, the enzyme expression was decreased, leading to consequential changes in both hormone production and testicular function in a multigenerational fashion (Huang et al., 2020).

## 5 Conclusion

Cadmium exposure, particularly during pregnancy, has been shown to induce many teratogenic effects and program the offspring to develop diseases later in life. Epigenetic alterations have been reported within cord blood, placenta, and fetal tissue upon cadmium exposure, leading to pregnancy complications, such as preeclampsia, and poor fetal growth. Sex-specific effects on epigenetic mechanisms are also observed, as the male offspring generally experience hypermethylation of genes, while the females show increased DNA hypomethylation, and many of the epigenetic alterations viewed in the fetal and neonatal stages persist into childhood. Interestingly, recent research has found that epigenetic alterations within germ cells pose multigenerational effects, in which adverse effects on the reproductive system were observed into the F2 offspring. However, there are limitations to the current knowledge. Comparing the studies, the experimental designs differ quite drastically, as cadmium is provided at different time points of pregnancy. Therefore, the results may differ between studies, and are not able to be accurately compared to draw conclusions. Additionally, most studies were focused on revealing genome-wide epigenetic changes, and the key genes that are adversely affected by epigenetic alterations are not clear. Further research on how the observed epigenetic changes influence gene expression, provoking disease development is warranted. More important, the selected epigenetic alterations and their associated genes should be validated in rigorously designed experiments. The fast developing gene editing technology has provided the possibility to introduce targeted epigenetic changes into animal models for verifying their roles in regulating gene expression and their multigenerational effects for disease development. Another limitation of currect research is that many of these studies observed epigenetic alterations in embryonic and fetal tissues, and discussed the potential for disease later in life, but these implications have yet to be investigated. Constructing experimental designs, in which the prenatal cadmium exposed offspring undergoes follow-up examination later in their life, either with or without continued cadmium treatment, is coveted for the advancement of the field. Moreover, information on the transgenerational effect of prenatal cadmium exposure on epigenetic alterations is lacking and warrants future research. Nevertheless, these results highlight cadmium’s role in epigenetic programming within the prenatal period and its impact on offspring health and disease development later in life.

## References

[B1] Ahir B. K. Elias N. M. Lakka S. S. (2017). SPARC overexpression alters microRNA expression profiles involved in tumor progression. Genes & cancer. 8, 453–471. 10.18632/genesandcancer.130 28435518 PMC5396623

[B2] Aoshima K. (1987). Epidemiology of renal tubular dysfunction in the inhabitants of a cadmium-polluted area in the Jinzu River basin in Toyama Prefecture. Tohoku J. Exp. Med. 152, 151–172. 10.1620/tjem.152.151 2442848

[B3] Awamleh Z. Gloor G. B. Han V. K. (2019). Placental microRNAs in pregnancies with early onset intrauterine growth restriction and preeclampsia: Potential impact on gene expression and pathophysiology. BMC Med. Genomics 12, 91–10. 10.1186/s12920-019-0548-x 31248403 PMC6598374

[B4] Awamleh Z. Han V. K. (2020). Potential pathophysiological role of microRNA 193b-5p in human placentae from pregnancies complicated by preeclampsia and intrauterine growth restriction. Mol. Biol. Rep. 47, 6531–6544. 10.1007/s11033-020-05705-y 32803505

[B5] Awe S. Renkawitz-Pohl R. (2010). Histone H4 acetylation is essential to proceed from a histone-to a protamine-based chromatin structure in spermatid nuclei of *Drosophila melanogaster* . Syst. Biol. reproductive Med. 56 (1), 44–61. 10.3109/19396360903490790 20170286

[B6] Banzato T. P. Godinho A. F. da Silva Zacarin Mathias E. C. Perobelli J. E. Fernandez C. D. B. (2012). Sperm quality in adult male rats exposed to cadmium *in utero* and lactation. J. Toxicol. Environ. Health, Part A. 75, 1047–1058. 10.1080/15287394.2012.697831 22852854

[B7] Barker D. J. Eriksson J. G. Forsén T. Osmond C. (2002). Fetal origins of adult disease: Strength of effects and biological basis. Int. J. Epidemiol. 31, 1235–1239. 10.1093/ije/31.6.1235 12540728

[B8] Bauer U. M. Körten M. Diller G. Helm P. Baumgartner H. Ewert P. (2019). Cardiovascular risk factors in adults with congenital heart defects—recognised but not treated? An analysis of the German national register for congenital heart defects. Int. J. Cardiol. 277, 79–84. 10.1016/j.ijcard.2018.08.009 30100225

[B9] Berger S. L. (2007). The complex language of chromatin regulation during transcription. Nature 447, 407–412. 10.1038/nature05915 17522673

[B10] Bokma J. P. Zegstroo I. Kuijpers J. M. Konings T. C. van Kimmenade R. R. van Melle J. P. (2018). Factors associated with coronary artery disease and stroke in adults with congenital heart disease. Heart 104, 574–580. 10.1136/heartjnl-2017-311620 28847851

[B11] Brooks S. A. Fry R. C. (2017). Cadmium inhibits placental trophoblast cell migration via miRNA regulation of the transforming growth factor beta (TGF-β) pathway. Food Chem. Toxicol. 109, 721–726. 10.1016/j.fct.2017.07.059 28774740 PMC5656529

[B12] Castillo P. Ibáñez F. Guajardo A. Llanos M. N. Ronco A. M. (2012). Impact of cadmium exposure during pregnancy on hepatic glucocorticoid receptor methylation and expression in rat fetus. Santiago, Chile: PLOS ONE. 10.1371/journal.pone.0044139 PMC3434215 22957049

[B13] Chatzi L. Ierodiakonou D. Margetaki K. Vafeiadi M. Chalkiadaki G. Roumeliotaki T. (2019). Associations of prenatal exposure to cadmium with child growth, obesity, and cardiometabolic traits. Am. J. Epidemiol. 188, 141–150. 10.1093/aje/kwy216 30252047 PMC8045476

[B14] Cowley M. Skaar D. A. Jima D. D. Maguire R. L. Hudson K. M. Park S. S. (2018). Effects of cadmium exposure on DNA methylation at imprinting control regions and genome-wide in mothers and newborn children. Environ. Health Perspect. 126, 037003. 10.1289/EHP2085 29529597 PMC6071808

[B15] Deng Y. Wu X. (2000). Peg3/Pw1 promotes p53-mediated apoptosis by inducing Bax translocation from cytosol to mitochondria. Proc. Natl. Acad. Sci. 97, 12050–12055. 10.1073/pnas.97.22.12050 11050235 PMC17292

[B16] Edwards M. (2017). The barker hypothesis. Handbook of famine, starvation, and nutrient deprivation: From Biology to policy. Switzerland: Springer, 191–211.

[B17] Engström A. Michaëlsson K. Vahter M. Julin B. Wolk A. Åkesson A. (2012). Associations between dietary cadmium exposure and bone mineral density and risk of osteoporosis and fractures among women. Bone 50, 1372–1378. 10.1016/j.bone.2012.03.018 22465267

[B18] Everson T. M. Armstrong D. A. Jackson B. P. Green B. B. Karagas M. R. Marsit C. J. (2016). Maternal cadmium, placental PCDHAC1, and fetal development. Reprod. Toxicol. 65, 263–271. 10.1016/j.reprotox.2016.08.011 27544570 PMC5226342

[B19] Gliga A. Igra A. Hellberg A. Engström K. Raqib R. Rahman A. (2022). Maternal exposure to cadmium during pregnancy is associated with changes in DNA methylation that are persistent at 9 years of age. Environ. Int. 163, 107188. 10.1016/j.envint.2022.107188 35334376

[B20] Hackett J. A. Sengupta R. Zylicz J. J. Murakami K. Lee C. Down T. A. (2013). Germline DNA demethylation dynamics and imprint erasure through 5-hydroxymethylcytosine. Science 339, 448–452. 10.1126/science.1229277 23223451 PMC3847602

[B21] Hayder H. O’Brien J. Nadeem U. Peng C. (2018). MicroRNAs: Crucial regulators of placental development. Reproduction 155, R259-R271–71. 10.1530/REP-17-0603 29615475

[B22] He L. Girijashanker K. Dalton T. P. Reed J. Li H. Soleimani M. (2006). ZIP8, member of the solute-carrier-39 (SLC39) metal-transporter family: Characterization of transporter properties. Mol. Pharmacol. 70, 171–180. 10.1124/mol.106.024521 16638970

[B23] Heard E. Martienssen R. A. (2014). Transgenerational epigenetic inheritance: Myths and mechanisms. Cell 157, 95–109. 10.1016/j.cell.2014.02.045 24679529 PMC4020004

[B24] Huang Y. Zhu J. Li H. Wang W. Li Y. Yang X. (2020). Cadmium exposure during prenatal development causes testosterone disruption in multigeneration via SF-1 signaling in rats. Food Chem. Toxicol. 135, 110897. 10.1016/j.fct.2019.110897 31654709

[B25] Hung T. Chen S. (2018). Risk of abnormal fetal growth in women with early-and late-onset preeclampsia. Pregnancy Hypertens. 12, 201–206. 10.1016/j.preghy.2017.09.003 29104027

[B26] Huntriss J. Epigenetic reprogramming in the embryo. InEpigenetics and reproductive health 2021. Cambridge, Massachusetts: Academic Press, 97–116.

[B27] Iijima K. Otake T. Yoshinaga J. Ikegami M. Suzuki E. Naruse H. (2007). Cadmium, lead, and selenium in cord blood and thyroid hormone status of newborns. Biol. Trace Elem. Res. 119, 10–18. 10.1007/s12011-007-0057-1 17914214

[B28] Ishitobi H. Mori K. Yoshida K. Watanabe C. (2007). Effects of perinatal exposure to low-dose cadmium on thyroid hormone-related and sex hormone receptor gene expressions in brain of offspring. Neurotoxicology 28, 790–797. 10.1016/j.neuro.2007.02.007 17408746

[B29] Jackson T. W. Baars O. Belcher S. M. (2022). Gestational Cd exposure in the CD-1 mouse sex-specifically disrupts essential metal ion homeostasis. Toxicol. Sci. 187, 254–266. 10.1093/toxsci/kfac027 35212737 PMC9154225

[B30] Järup L. Berglund M. Elinder C. G. Nordberg G. Vanter M. (1998). Health effects of cadmium exposure–a review of the literature and a risk estimate. Scand. J. Work Environ. Health. 24, 1–51. 9569444

[B31] Jin X. Tian X. Liu Z. Hu H. Li X. Deng Y. (2016). Maternal exposure to arsenic and cadmium and the risk of congenital heart defects in offspring. Reprod. Toxicol. 59, 109–116. 10.1016/j.reprotox.2015.12.007 26743994

[B32] Johri N. Jacquillet G. Unwin R. (2010). Heavy metal poisoning: The effects of cadmium on the kidney. Biometals 23, 783–792. 10.1007/s10534-010-9328-y 20354761

[B33] Joubert B. R. Håberg S. E. Nilsen R. M. Wang X. Vollset S. E. Murphy S. K. (2012). 450K epigenome-wide scan identifies differential DNA methylation in newborns related to maternal smoking during pregnancy. Environ. Health Perspect. 120, 1425–1431. 10.1289/ehp.1205412 22851337 PMC3491949

[B34] Kippler M. Engström K. Mlakar S. J. Bottai M. Ahmed S. Hossain M. B. (2013). Sex-specific effects of early life cadmium exposure on DNA methylation and implications for birth weight. Epigenetics 8, 494–503. 10.4161/epi.24401 23644563 PMC3741219

[B35] Kippler M. Wagatsuma Y. Rahman A. Nermell B. Persson L. Raqib R. (2012). Environmental exposure to arsenic and cadmium during pregnancy and fetal size: A longitudinal study in rural Bangladesh. Reprod. Toxicol. 34, 504–511. 10.1016/j.reprotox.2012.08.002 22985739

[B36] Koppes E. Himes K. P. Chaillet J. R. (2015). Partial loss of genomic imprinting reveals important roles for Kcnq1 and Peg10 imprinted domains in placental development. PloS one 10, e0135202. 10.1371/journal.pone.0135202 26241757 PMC4524636

[B37] Kotlabova K. Doucha J. Hromadnikova I. (2011). Placental-specific microRNA in maternal circulation–identification of appropriate pregnancy-associated microRNAs with diagnostic potential. J. Reprod. Immunol. 89, 185–191. 10.1016/j.jri.2011.02.006 21513988

[B38] Li S. Chen M. Li Y. Tollefsbol T. O. (2019). Prenatal epigenetics diets play protective roles against environmental pollution. Clin. epigenetics 11 (1), 82–31. 10.1186/s13148-019-0659-4 31097039 PMC6524340

[B39] Li X. Wang L. Li Y. Fu J. Zhen L. Yang Q. (2016). Tyrosine phosphorylation of dihydrolipoamide dehydrogenase as a potential cadmium target and its inhibitory role in regulating mouse sperm motility. Toxicology 357, 52–64. 10.1016/j.tox.2016.06.003 27289041

[B40] Liu J. Liao J. Zhang C. Zeng L. Zong C. Lv Y. (2021). The role of miRNAs in regulating the effect of prenatal cadmium exposure on ovarian granulosa cells in a transgenerational manner in female rats. Food Chem. Toxicol. 150, 112062. 10.1016/j.fct.2021.112062 33652105

[B41] Liu J. Zeng L. Zhuang S. Zhang C. Li Y. Zhu J. (2020). Cadmium exposure during prenatal development causes progesterone disruptors in multiple generations via steroidogenic enzymes in rat ovarian granulosa cells. Ecotoxicol. Environ. Saf. 201, 110765. 10.1016/j.ecoenv.2020.110765 32497815

[B42] Manikkam M. Guerrero-Bosagna C. Tracey R. Haque M. M. Skinner M. K. (2012). Transgenerational actions of environmental compounds on reproductive disease and identification of epigenetic biomarkers of ancestral exposures. PloS one 7, e31901. 10.1371/journal.pone.0031901 22389676 PMC3289630

[B43] McKinney D. Boyd H. Langager A. Oswald M. Pfister A. Warshak C. R., The impact of fetal growth restriction on latency in the setting of expectant management of preeclampsia. Obstet. Gynecol. 2016;214:395. 10.1016/j.ajog.2015.12.050 26767794

[B44] Mikolić A. Piasek M. Sulimanec Grgec A. Varnai V. M. Stasenko S. Kralik Oguić S. (2015). Oral cadmium exposure during rat pregnancy: Assessment of transplacental micronutrient transport and steroidogenesis at term. J. Appl. Toxicol. 35, 508–519. 10.1002/jat.3055 25256609

[B45] Mohanty A. F. Farin F. M. Bammler T. K. MacDonald J. W. Afsharinejad Z. Burbacher T. M. (2015). Infant sex-specific placental cadmium and DNA methylation associations. Environ. Res. 138, 74–81. 10.1016/j.envres.2015.02.004 25701811 PMC4385453

[B46] Mouillet J. Ouyang Y. Coyne C. B. Sadovsky Y. (2015). MicroRNAs in placental health and disease. Obstet. Gynecol. 213, S163–S172. 10.1016/j.ajog.2015.05.057 PMC4592520 26428496

[B47] Östling H. Kruse R. Helenius G. Lodefalk M. (2019). Placental expression of microRNAs in infants born small for gestational age. Placenta 81, 46–53. 10.1016/j.placenta.2019.05.001 31138431

[B48] Ou Y. Bloom M. S. Nie Z. Han F. Mai J. Chen J. (2017). Associations between toxic and essential trace elements in maternal blood and fetal congenital heart defects. Environ. Int. 106, 127–134. 10.1016/j.envint.2017.05.017 28645012

[B49] Pan Y. Robertson G. Pedersen L. Lim E. Hernandez-Herrera A. Rowat A. C. (2016). miR-509-3p is clinically significant and strongly attenuates cellular migration and multi-cellular spheroids in ovarian cancer. Oncotarget 7, 25930–25948. 10.18632/oncotarget.8412 27036018 PMC5041955

[B50] Park J. Kim J. Kim E. Won S. Kim W. J. (2022). Association between prenatal cadmium exposure and cord blood DNA methylation. Environ. Res. 212, 113268. 10.1016/j.envres.2022.113268 35405126

[B51] Pizzol M. Smart J. C. Thomsen M. (2014). External costs of cadmium emissions to soil: A drawback of phosphorus fertilizers. J. Clean. Prod. 84, 475–483. 10.1016/j.jclepro.2013.12.080

[B52] Ramos-Ruiz A. Wilkening J. V. Field J. A. Sierra-Alvarez R. (2017). Leaching of cadmium and tellurium from cadmium telluride (CdTe) thin-film solar panels under simulated landfill conditions. J. Hazard. Mat. 336, 57–64. 10.1016/j.jhazmat.2017.04.052 PMC5607867 28472709

[B53] Rana S. Lemoine E. Granger J. P. Karumanchi S. A. (2019). Preeclampsia: Pathophysiology, challenges, and perspectives. Circ. Res. 124, 1094–1112. 10.1161/CIRCRESAHA.118.313276 30920918

[B54] Rossant J. Cross J. C. (2001). Placental development: Lessons from mouse mutants. Nat. Rev. Genet. 2, 538–548. 10.1038/35080570 11433360

[B55] Ryznar R. J. Phibbs L. Van Winkle L. J. (2021). Epigenetic modifications at the center of the barker hypothesis and their transgenerational implications. Int. J. Environ. Res. Public Health 18, 12728. 10.3390/ijerph182312728 34886453 PMC8656758

[B56] Samuel J. B. Stanley J. A. Princess R. A. Shanthi P. Sebastian M. S. (2011). Gestational cadmium exposure-induced ovotoxicity delays puberty through oxidative stress and impaired steroid hormone levels. J. Med. Toxicol. 7, 195–204. 10.1007/s13181-011-0143-9 21373971 PMC3550211

[B57] Sanders A. Smeester L. Rojas D. DeBussycher T. Wu M. Wright F. (2014). Cadmium exposure and the epigenome: Exposure-associated patterns of DNA methylation in leukocytes from mother-baby pairs. Epigenetics 9, 212–221. 10.4161/epi.26798 24169490 PMC3962531

[B58] Santos F. Peters A. H. Otte A. P. Reik W. Dean W. (2005). Dynamic chromatin modifications characterise the first cell cycle in mouse embryos. Dev. Biol. 280 (1), 225–236. 10.1016/j.ydbio.2005.01.025 15766761

[B59] Satarug S. Vesey D. A. Gobe G. C. (2017). Kidney cadmium toxicity, diabetes and high blood pressure: The perfect storm. Tohoku J. Exp. Med. 241, 65–87. 10.1620/tjem.241.65 28132967

[B60] Satoh M. Koyama H. Kaji T. Kito H. Tohyama C. (2002). Perspectives on cadmium toxicity research. Tohoku J. Exp. Med. 196, 23–32. 10.1620/tjem.196.23 12498323

[B61] Seisenberger S. Andrews S. Krueger F. Arand J. Walter J. Santos F. (2012). The dynamics of genome-wide DNA methylation reprogramming in mouse primordial germ cells. Mol. Cell. 48, 849–862. 10.1016/j.molcel.2012.11.001 23219530 PMC3533687

[B62] Simmers M. D. Hudson K. M. Baptissart M. Cowley M. (2022). Epigenetic control of the imprinted growth regulator Cdkn1c in cadmium-induced placental dysfunction. Epigenetics, 1–17. 10.1080/15592294.2022.2088173 PMC10989690 35770551

[B63] Sinclair K. D. Lea R. G. Rees W. D. Young L. E. (2007). The developmental origins of health and disease: Current theories and epigenetic mechanisms. Soc. Reproduction Fertil. Suppl. 64, 425–443. 10.5661/rdr-vi-425 17491163

[B64] Stasenko S. Bradford E. M. Piasek M. Henson M. C. Varnai V. M. Jurasović J. (2010). Metals in human placenta: Focus on the effects of cadmium on steroid hormones and leptin. J. Appl. Toxicol. Int. J. 30, 242–253. 10.1002/jat.1490 19847775

[B65] Su Z. Chen D. Zhang E. Li Y. Yu Z. Shi M. (2015). MicroRNA-509-3p inhibits cancer cell proliferation and migration by targeting the mitogen-activated protein kinase kinase kinase 8 oncogene in renal cell carcinoma. Mol. Med. Rep. 12, 1535–1543. 10.3892/mmr.2015.3498 25815776

[B66] Takahashi Y. Valencia M. M. Yu Y. Ouchi Y. Takahashi K. Shokhirev M. N. 2023. Transgenerational inheritance of acquired epigenetic signatures at CpG islands in mice. Cell. 186, 731. 10.1016/j.cell.2022.12.047 36754048

[B67] Tehrani J. M. Kennedy E. Tung P. W. Burt A. Hermetz K. Punshon T. (2022). Human placental microRNAs dysregulated by cadmium exposure predict neurobehavioral outcomes at birth. Pediatr. Res., 1–9. 10.1038/s41390-022-02201-w PMC9884320 35906307

[B68] Tenório M. B. Ferreira R. C. Moura F. A. Bueno N. B. Goulart M. O. F. (2019). Cross-talk between oxidative stress and inflammation in preeclampsia. Oxidative medicine and cellular longevity. Maceio, Brazil: Hindawi (Oxidative Medicine and Cellular Longevity). 10.1155/2019/8238727 PMC6875353 31781353

[B69] Vaiserman A. (2014). Early-life exposure to endocrine disrupting chemicals and later-life health outcomes: An epigenetic bridge? Aging Dis. 5, 419–429. 10.14336/AD.2014.0500419 25489493 PMC4249811

[B70] Vidal A. C. Semenova V. Darrah T. Vengosh A. Huang Z. King K. (2015). Maternal cadmium, iron and zinc levels, DNA methylation and birth weight. BMC Pharmacol. Toxicol. 16, 20–29. 10.1186/s40360-015-0020-2 26173596 PMC4502530

[B71] Vilahur N. Vahter M. Broberg K. (2015). The epigenetic effects of prenatal cadmium exposure. Curr. Environ. health Rep. 2, 195–203. 10.1007/s40572-015-0049-9 25960943 PMC4417128

[B72] Waalkes M. P. (2003). Cadmium carcinogenesis. Mutat. Research/Fundamental Mol. Mech. Mutagen. 533, 107–120. 10.1016/j.mrfmmm.2003.07.011 14643415

[B73] Wang H. Wang Y. Bo Q. Ji Y. Liu L. Hu Y. (2016). Maternal cadmium exposure reduces placental zinc transport and induces fetal growth restriction in mice. Reprod. Toxicol. 63, 174–182. 10.1016/j.reprotox.2016.06.010 27319394

[B74] Wang L. Li Y. Fu J. Zhen L. Zhao N. Yang Q. (2016). Cadmium inhibits mouse sperm motility through inducing tyrosine phosphorylation in a specific subset of proteins. Reprod. Toxicol. 63, 96–106. 10.1016/j.reprotox.2016.05.018 27233480

[B75] Wier P. J. Miller R. K. Maulik D. di Sant'Agnese P. A. (1990). Toxicity of cadmium in the perfused human placenta. Toxicol. Appl. Pharmacol. 105, 156–171. 10.1016/0041-008x(90)90367-4 2392803

[B76] Woods L. Perez-Garcia V. Hemberger M. (2018). Regulation of placental development and its impact on fetal growth—New insights from mouse models. Front. Endocrinol. 9, 570. 10.3389/fendo.2018.00570 PMC6170611 30319550

[B77] Xie Z. Dai J. Dai L. Tan M. Cheng Z. Wu Y. (2012). Lysine succinylation and lysine malonylation in histones. Mol. Cell. Proteomics 11 (5), 100–107. 10.1074/mcp.M111.015875 22389435 PMC3418837

[B78] Xu P. Wu Z. Xi Y. Wang L. (2016). Epigenetic regulation of placental glucose transporters mediates maternal cadmium-induced fetal growth restriction. Toxicology 372, 34–41. 10.1016/j.tox.2016.10.011 27931521

[B79] Xu P. Wu Z. Yang W. Wang L. (2017). Dysregulation of DNA methylation and expression of imprinted genes in mouse placentas of fetal growth restriction induced by maternal cadmium exposure. Toxicology 390, 109–116. 10.1016/j.tox.2017.08.003 28823913

[B80] Yang K. Julan L. Rubio F. Sharma A. Guan H. (2006). Cadmium reduces 11β-hydroxysteroid dehydrogenase type 2 activity and expression in human placental trophoblast cells. Am. J. Physiology-Endocrinology Metabolism 290, E135–E142. 10.1152/ajpendo.00356.2005 16144812

[B81] Yang Q. Li P. Wen Y. Li S. Chen J. Liu X. (2018). Cadmium inhibits lysine acetylation and succinylation inducing testicular injury of mouse during development. Toxicol. Lett. 291, 112–120. 10.1016/j.toxlet.2018.04.005 29653258

[B82] Yi S. Xiong Y. Zhu H. Dai L. Cao X. Liu W. (2021). Environmental cadmium exposure during pregnancy causes diabetes-like phenotypes in mouse offspring: Association with oxidative stress in the fetal liver. Sci. Total Environ. 777, 146006. 10.1016/j.scitotenv.2021.146006 33677283

[B83] Zhang G. Wang H. Hu J. Guo M. Wang Y. Zhou Y. (2016). Cadmium-induced neural tube defects and fetal growth restriction: Association with disturbance of placental folate transport. Toxicol. Appl. Pharmacol. 306, 79–85. 10.1016/j.taap.2016.07.007 27417525

[B84] Zhang Y. Xu X. Chen A. Davuljigari C. B. Zheng X. Kim S. S. (2018). Maternal urinary cadmium levels during pregnancy associated with risk of sex-dependent birth outcomes from an e-waste pollution site in China. Reprod. Toxicol. 75, 49–55. 10.1016/j.reprotox.2017.11.003 29154917

[B85] Zhou X. Li Q. Xu J. Zhang X. Zhang H. Xiang Y. (2016). The aberrantly expressed miR-193b-3p contributes to preeclampsia through regulating transforming growth factor-β signaling. Sci. Rep. 6, 19910–19913. 10.1038/srep19910 26822621 PMC4731805

[B86] Zhu J. Huang Z. Yang F. Zhu M. Cao J. Chen J. (2021). Cadmium disturbs epigenetic modification and induces DNA damage in mouse preimplantation embryos. Ecotoxicol. Environ. Saf. 219, 112306. 10.1016/j.ecoenv.2021.112306 33984557

